# 4-(5-Hy­droxy­meth­yl-2-meth­oxy­phen­oxy)benzoic acid

**DOI:** 10.1107/S1600536811012190

**Published:** 2011-04-07

**Authors:** Yanyan Niu, Bo Wu

**Affiliations:** a School of Chemistry and Chemical Engineering, Shandong University, Jinan, 250100, People’s Republic of China

## Abstract

The title compound, C_15_H_14_O_5_, crystallizes with two independent mol­ecules in the asymmetric unit in which the benzene rings are inclined at dihedral angles of 79.4 (1) and 84.2 (1)°. In the crystal, inter­molecular O—H⋯O hydrogen bonds link the mol­ecules into double chains propagating in [001].

## Related literature

For the bioactivity of diphenyl ether derivatives, see: Asakawa (2001 ▶); Hua *et al.* (2009 ▶); Kini *et al.*(2009 ▶). For background to Ullman coupling, see: Bringmann *et al.* (1990 ▶).
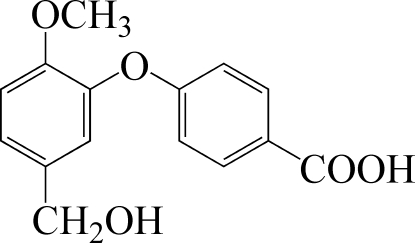


## Experimental

### 

#### Crystal data

C_15_H_14_O_5_
*M* *_r_* = 274.26
Triclinic, 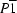

*a* = 10.5420 (15) Å
*b* = 10.6153 (15) Å
*c* = 12.8070 (18) Å
α = 78.024 (2)°
β = 75.184 (2)°
γ = 88.451 (2)°
*V* = 1354.9 (3) Å^3^
*Z* = 4
Mo *K*α radiation
μ = 0.10 mm^−1^
*T* = 273 K
0.13 × 0.12 × 0.10 mm

#### Data collection

Bruker APEXII CCD diffractometer
Absorption correction: multi-scan (*SADABS*; Bruker, 2007 ▶) *T*_min_ = 0.987, *T*_max_ = 0.990
5794 measured reflections
3874 independent reflections
3154 reflections with *I* > 2σ(*I*)
*R*_int_ = 0.015
θ_max_ = 23.3°

#### Refinement

*R*[*F* ^2^ > 2σ(*F* ^2^)] = 0.045
*wR*(*F* ^2^) = 0.141
*S* = 1.02
3874 reflections
365 parameters
H-atom parameters constrained
Δρ_max_ = 0.22 e Å^−3^
Δρ_min_ = −0.19 e Å^−3^

### 

Data collection: *APEX2* (Bruker, 2007 ▶); cell refinement: *SAINT* (Bruker, 2007 ▶); data reduction: *SAINT*; program(s) used to solve structure: *SHELXS97* (Sheldrick, 2008 ▶); program(s) used to refine structure: *SHELXL97* (Sheldrick, 2008 ▶); molecular graphics: *SHELXTL* (Sheldrick, 2008 ▶); software used to prepare material for publication: *SHELXTL*.

## Supplementary Material

Crystal structure: contains datablocks I, global. DOI: 10.1107/S1600536811012190/cv5067sup1.cif

Structure factors: contains datablocks I. DOI: 10.1107/S1600536811012190/cv5067Isup2.hkl

Additional supplementary materials:  crystallographic information; 3D view; checkCIF report

## Figures and Tables

**Table 1:** Hydrogen-bond geometry (Å, °)

*D*—H⋯*A*	*D*—H	H⋯*A*	*D*⋯*A*	*D*—H⋯*A*
O9—H9⋯O1	0.82	1.80	2.620 (2)	175
O4—H4⋯O6	0.82	1.84	2.652 (2)	1670
O6—H6⋯O10^i^	0.82	2.01	2.791 (3)	159
O1—H1⋯O5^ii^	0.82	1.89	2.706 (2)	172

Symmetry codes: (i) 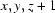; (ii) 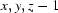.

**Fig. 1.**
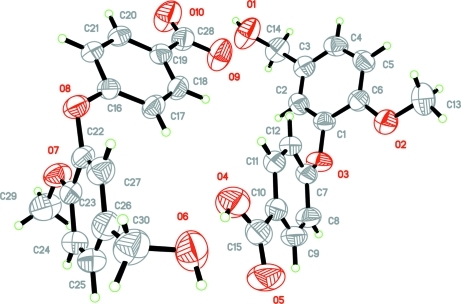
Two independent molecules of (I) showing the atomic numbering and 50% probabilty displacement ellipsoids.

## supplementary crystallographic information

### Comment

The diphenyl ether analogs existing in many natural products exhibit various bioactivities, such as antitubercular (Kini *et al.*, 2009), antibacterial (Hua *et al.*, 2009), and cytotoxic (Asakawa, 2001) activities. Most of the diphenyl ethers were synthesized by Ullman coupling (Bringmann *et al.*, 1990), using Cu complexes as catalysts. Herewith we present the title compound (I) - a new derivative of diphenyl ether.

The asymmetric unit of (I) contains two independent molecules (Fig. 1). In the independent molecules, two benzene rings form the dihedral angles of 79.4 (1) and 84.2 (1)°, respectively. In the crystal structure, O—H···O hydrogen bonds (Table 1) link the molecules into doubled chains propagated in [001].

### Experimental

(3-Bromo-4-methoxyphenyl)methanol (5.00 g, 23.04 mmol), and methyl 4-hydroxybenzoate (3.50 g, 23.04 mmol), potassium carbonate (3.17 g, 46.08 mmol), and cupric oxide (0.18 g, 2.25 mmol) in pyridine (20 ml) were added in flask and the mixture was stirred under reflux for 12 h. The pyridine was distilled *in vacuo* and the residue was extracted with CH_2_Cl_2_(3*30 ml). The solution was concentrated and the residue was purified by flash column chromatograph on Al_2_O_3_, The yield of the coupling product was 4.35 g (65%) as white solid. The coupling products was hydrolyzed with 20% NaOH aq and then acidified to pH=6.0 with 1*M* HCl. The final product was extracted with CH_2_Cl_2_ (3*20 ml) and obtained the white solid by vacuum distillation. (4.11 g, 99%). The colourless crystals suitable for an X-ray diffraction experiment were obtained by cystal growth from ethanol.

### Refinement

All the H atoms were located in difference maps; then placed in idealized positions [C—H 0.93–0.97 Å; O—H 0.82 Å] and treated as riding atoms, with *U*_iso_(H) = 1.2–1.5 *U*_eq_ of the parent atom.

### Crystal data

**Table:**

C_15_H_14_O_5_	*Z* = 4
*M**_r_* = 274.26	*F*(000) = 576
Triclinic, *P*1	*D*_x_ = 1.345 Mg m^−^^3^
Hall symbol: -P 1	Mo *K*α radiation, λ = 0.71073 Å
*a* = 10.5420 (15) Å	Cell parameters from 2550 reflections
*b* = 10.6153 (15) Å	θ = 2.8–23.3°
*c* = 12.8070 (18) Å	µ = 0.10 mm^−^^1^
α = 78.024 (2)°	*T* = 273 K
β = 75.184 (2)°	Block, colourless
γ = 88.451 (2)°	0.13 × 0.12 × 0.10 mm
*V* = 1354.9 (3) Å^3^	

### Data collection

**Table:**

Bruker APEXII CCD diffractometer	3874 independent reflections
Radiation source: fine-focus sealed tube	3154 reflections with *I* > 2σ(*I*)
graphite	*R*_int_ = 0.015
phi and ω scans	θ_max_ = 23.3°, θ_min_ = 2.0°
Absorption correction: multi-scan (*SADABS*; Bruker, 2007)	*h* = −11→11
*T*_min_ = 0.987, *T*_max_ = 0.990	*k* = −11→5
5794 measured reflections	*l* = −14→13

### Refinement

**Table:**

Refinement on *F*^2^	Primary atom site location: structure-invariant direct methods
Least-squares matrix: full	Secondary atom site location: difference Fourier map
*R*[*F*^2^ > 2σ(*F*^2^)] = 0.045	Hydrogen site location: inferred from neighbouring sites
*wR*(*F*^2^) = 0.141	H-atom parameters constrained
*S* = 1.02	*w* = 1/[σ^2^(*F*_o_^2^) + (0.093*P*)^2^ + 0.1798*P*] where *P* = (*F*_o_^2^ + 2*F*_c_^2^)/3
3874 reflections	(Δ/σ)_max_ < 0.001
365 parameters	Δρ_max_ = 0.22 e Å^−^^3^
0 restraints	Δρ_min_ = −0.19 e Å^−^^3^

### Special details

**Table:**

Geometry. All e.s.d.'s (except the e.s.d. in the dihedral angle between two l.s. planes) are estimated using the full covariance matrix. The cell e.s.d.'s are taken into account individually in the estimation of e.s.d.'s in distances, angles and torsion angles; correlations between e.s.d.'s in cell parameters are only used when they are defined by crystal symmetry. An approximate (isotropic) treatment of cell e.s.d.'s is used for estimating e.s.d.'s involving l.s. planes.
Refinement. Refinement of *F*^2^ against ALL reflections. The weighted *R*-factor *wR* and goodness of fit *S* are based on *F*^2^, conventional *R*-factors *R* are based on *F*, with *F* set to zero for negative *F*^2^. The threshold expression of *F*^2^ > σ(*F*^2^) is used only for calculating *R*-factors(gt) *etc*. and is not relevant to the choice of reflections for refinement. *R*-factors based on *F*^2^ are statistically about twice as large as those based on *F*, and *R*- factors based on ALL data will be even larger.

### Fractional atomic coordinates and isotropic or equivalent isotropic displacement parameters (Å^2^)

**Table:**

	*x*	*y*	*z*	*U*_iso_*/*U*_eq_	
O8	0.45863 (12)	0.12659 (13)	0.65556 (10)	0.0507 (4)	
O7	0.68968 (14)	0.00872 (15)	0.63970 (12)	0.0595 (4)	
C22	0.52664 (18)	0.12698 (18)	0.73608 (16)	0.0447 (5)	
C16	0.51038 (17)	0.20109 (18)	0.55175 (15)	0.0424 (5)	
C19	0.60706 (19)	0.33828 (19)	0.33809 (16)	0.0480 (5)	
C20	0.5219 (2)	0.2327 (2)	0.35983 (17)	0.0500 (5)	
H20A	0.4978	0.2079	0.3019	0.060*	
C23	0.64416 (19)	0.06201 (18)	0.73023 (16)	0.0470 (5)	
C21	0.47319 (19)	0.16482 (19)	0.46587 (16)	0.0475 (5)	
H21A	0.4156	0.0949	0.4799	0.057*	
C27	0.4736 (2)	0.1865 (2)	0.82238 (16)	0.0510 (5)	
H27A	0.3956	0.2301	0.8245	0.061*	
C24	0.7062 (2)	0.0578 (2)	0.81386 (18)	0.0566 (6)	
H24A	0.7845	0.0148	0.8119	0.068*	
C17	0.5919 (2)	0.30742 (19)	0.53229 (17)	0.0511 (5)	
H17A	0.6142	0.3332	0.5905	0.061*	
O10	0.6491 (2)	0.36489 (19)	0.14387 (14)	0.0957 (7)	
O9	0.7344 (2)	0.50767 (18)	0.21132 (13)	0.0866 (6)	
H9	0.7690	0.5355	0.1457	0.130*	
C18	0.6399 (2)	0.3752 (2)	0.42520 (16)	0.0520 (5)	
H18A	0.6952	0.4469	0.4116	0.062*	
C26	0.5356 (2)	0.1820 (2)	0.90646 (16)	0.0543 (5)	
C25	0.6514 (2)	0.1177 (2)	0.90050 (18)	0.0605 (6)	
H25A	0.6940	0.1144	0.9562	0.073*	
C28	0.6631 (2)	0.4042 (2)	0.22227 (18)	0.0590 (6)	
C29	0.8069 (3)	−0.0626 (3)	0.6332 (2)	0.0796 (8)	
H29A	0.8279	−0.0952	0.5665	0.119*	
H29B	0.7942	−0.1332	0.6957	0.119*	
H29C	0.8776	−0.0074	0.6331	0.119*	
O3	1.04180 (13)	0.76114 (15)	0.31213 (12)	0.0619 (4)	
C7	0.9680 (2)	0.70611 (19)	0.41650 (17)	0.0502 (5)	
O2	0.89962 (16)	0.97320 (16)	0.31548 (14)	0.0700 (5)	
O5	0.8207 (2)	0.5321 (2)	0.82261 (14)	0.0896 (6)	
C1	0.97799 (19)	0.8027 (2)	0.22881 (17)	0.0516 (5)	
C12	0.8381 (2)	0.66884 (19)	0.43978 (17)	0.0501 (5)	
H12A	0.7941	0.6824	0.3842	0.060*	
C10	0.8387 (2)	0.59194 (19)	0.63005 (17)	0.0524 (5)	
C11	0.7735 (2)	0.61085 (19)	0.54690 (17)	0.0503 (5)	
H11A	0.6860	0.5844	0.5632	0.060*	
C3	0.9363 (2)	0.7804 (2)	0.05822 (17)	0.0560 (6)	
C6	0.90656 (19)	0.9154 (2)	0.22828 (17)	0.0517 (5)	
C2	0.9932 (2)	0.7374 (2)	0.14517 (18)	0.0561 (5)	
H2A	1.0423	0.6632	0.1466	0.067*	
O4	0.65453 (19)	0.48294 (19)	0.75911 (13)	0.0810 (5)	
H4	0.6192	0.4596	0.8251	0.122*	
C8	1.0346 (2)	0.6871 (2)	0.4979 (2)	0.0612 (6)	
H8A	1.1225	0.7124	0.4811	0.073*	
C15	0.7709 (3)	0.5337 (2)	0.74642 (18)	0.0630 (6)	
C5	0.8493 (2)	0.9587 (2)	0.14235 (19)	0.0589 (6)	
H5A	0.8006	1.0331	0.1406	0.071*	
C9	0.9694 (2)	0.6303 (2)	0.60459 (19)	0.0616 (6)	
H9A	1.0137	0.6177	0.6599	0.074*	
C4	0.8646 (2)	0.8908 (2)	0.05883 (18)	0.0603 (6)	
H4A	0.8254	0.9206	0.0014	0.072*	
C14	0.9473 (3)	0.7054 (3)	−0.03082 (19)	0.0724 (7)	
H14A	0.9394	0.7625	−0.0984	0.087*	
H14B	1.0321	0.6655	−0.0453	0.087*	
C13	0.8180 (3)	1.0823 (2)	0.3227 (2)	0.0787 (7)	
H13A	0.8209	1.1140	0.3870	0.118*	
H13B	0.7294	1.0575	0.3279	0.118*	
H13C	0.8490	1.1487	0.2580	0.118*	
O6	0.5358 (2)	0.37953 (16)	0.96729 (14)	0.0863 (6)	
H6	0.5827	0.3883	1.0075	0.129*	
O1	0.8463 (2)	0.6102 (2)	0.00454 (13)	0.0946 (7)	
H1	0.8407	0.5796	−0.0479	0.142*	
C30	0.4822 (3)	0.2530 (3)	0.99757 (19)	0.0717 (7)	
H30A	0.3874	0.2556	1.0121	0.086*	
H30B	0.5041	0.2083	1.0646	0.086*	

### Atomic displacement parameters (Å^2^)

**Table:**

	*U*^11^	*U*^22^	*U*^33^	*U*^12^	*U*^13^	*U*^23^
O8	0.0490 (8)	0.0603 (9)	0.0454 (8)	−0.0110 (6)	−0.0185 (6)	−0.0068 (7)
O7	0.0618 (9)	0.0643 (9)	0.0656 (10)	0.0097 (7)	−0.0272 (7)	−0.0302 (8)
C22	0.0472 (11)	0.0465 (11)	0.0426 (11)	−0.0110 (9)	−0.0170 (9)	−0.0060 (9)
C16	0.0414 (10)	0.0445 (11)	0.0446 (11)	0.0036 (8)	−0.0165 (8)	−0.0102 (9)
C19	0.0531 (11)	0.0491 (11)	0.0458 (11)	0.0032 (9)	−0.0194 (9)	−0.0108 (9)
C20	0.0585 (12)	0.0534 (12)	0.0479 (12)	−0.0010 (10)	−0.0275 (10)	−0.0148 (10)
C23	0.0549 (12)	0.0412 (11)	0.0484 (12)	−0.0052 (9)	−0.0197 (9)	−0.0082 (9)
C21	0.0477 (11)	0.0470 (11)	0.0546 (13)	−0.0028 (9)	−0.0240 (9)	−0.0119 (10)
C27	0.0496 (11)	0.0546 (12)	0.0478 (12)	−0.0043 (9)	−0.0104 (9)	−0.0102 (10)
C24	0.0651 (13)	0.0525 (12)	0.0597 (13)	0.0017 (10)	−0.0315 (11)	−0.0090 (10)
C17	0.0613 (12)	0.0504 (12)	0.0479 (12)	−0.0092 (10)	−0.0214 (10)	−0.0136 (9)
O10	0.1400 (18)	0.1021 (15)	0.0491 (10)	−0.0394 (13)	−0.0320 (10)	−0.0091 (10)
O9	0.1231 (15)	0.0779 (12)	0.0519 (10)	−0.0380 (11)	−0.0120 (10)	−0.0062 (9)
C18	0.0616 (13)	0.0464 (11)	0.0518 (13)	−0.0090 (10)	−0.0193 (10)	−0.0111 (10)
C26	0.0656 (14)	0.0556 (13)	0.0401 (11)	−0.0127 (11)	−0.0113 (10)	−0.0075 (9)
C25	0.0786 (16)	0.0599 (14)	0.0505 (13)	−0.0066 (12)	−0.0334 (11)	−0.0063 (10)
C28	0.0713 (14)	0.0584 (14)	0.0496 (13)	−0.0051 (11)	−0.0208 (11)	−0.0089 (11)
C29	0.0760 (16)	0.0827 (18)	0.099 (2)	0.0262 (14)	−0.0367 (15)	−0.0474 (16)
O3	0.0482 (8)	0.0761 (10)	0.0612 (10)	0.0049 (7)	−0.0208 (7)	−0.0056 (8)
C7	0.0533 (12)	0.0481 (12)	0.0552 (13)	0.0113 (9)	−0.0229 (10)	−0.0143 (10)
O2	0.0745 (10)	0.0656 (10)	0.0815 (11)	0.0104 (8)	−0.0311 (9)	−0.0285 (9)
O5	0.1208 (15)	0.1074 (15)	0.0559 (10)	0.0156 (12)	−0.0428 (11)	−0.0273 (10)
C1	0.0435 (11)	0.0556 (13)	0.0555 (13)	−0.0004 (9)	−0.0175 (9)	−0.0046 (10)
C12	0.0549 (12)	0.0488 (11)	0.0534 (12)	0.0040 (9)	−0.0265 (10)	−0.0103 (10)
C10	0.0704 (14)	0.0436 (11)	0.0539 (13)	0.0145 (10)	−0.0288 (11)	−0.0196 (9)
C11	0.0600 (12)	0.0445 (11)	0.0537 (13)	0.0056 (9)	−0.0246 (10)	−0.0141 (10)
C3	0.0550 (12)	0.0602 (14)	0.0467 (12)	−0.0099 (10)	−0.0066 (10)	−0.0038 (10)
C6	0.0452 (11)	0.0513 (12)	0.0570 (13)	−0.0050 (9)	−0.0114 (9)	−0.0085 (10)
C2	0.0527 (12)	0.0523 (12)	0.0594 (14)	0.0043 (10)	−0.0105 (10)	−0.0084 (11)
O4	0.1012 (14)	0.0868 (13)	0.0534 (10)	−0.0133 (11)	−0.0265 (9)	−0.0004 (9)
C8	0.0554 (13)	0.0679 (14)	0.0722 (16)	0.0094 (11)	−0.0329 (12)	−0.0211 (12)
C15	0.0921 (18)	0.0545 (13)	0.0526 (14)	0.0174 (13)	−0.0313 (13)	−0.0201 (11)
C5	0.0561 (13)	0.0518 (13)	0.0648 (14)	0.0032 (10)	−0.0176 (11)	−0.0007 (11)
C9	0.0741 (15)	0.0658 (14)	0.0603 (15)	0.0166 (12)	−0.0390 (12)	−0.0221 (12)
C4	0.0563 (13)	0.0685 (15)	0.0512 (13)	−0.0053 (11)	−0.0170 (10)	0.0033 (11)
C14	0.0780 (16)	0.0839 (18)	0.0500 (13)	−0.0120 (14)	−0.0063 (11)	−0.0131 (12)
C13	0.0876 (18)	0.0606 (15)	0.0837 (18)	0.0078 (13)	−0.0080 (14)	−0.0234 (13)
O6	0.1443 (18)	0.0613 (11)	0.0554 (10)	−0.0033 (11)	−0.0256 (10)	−0.0161 (8)
O1	0.1292 (16)	0.1022 (15)	0.0485 (10)	−0.0476 (13)	−0.0084 (10)	−0.0180 (10)
C30	0.0852 (17)	0.0803 (17)	0.0516 (14)	−0.0086 (13)	−0.0136 (12)	−0.0216 (12)

### Geometric parameters (Å, °)

**Table:**

O8—C16	1.381 (2)	O2—C6	1.367 (3)
O8—C22	1.399 (2)	O2—C13	1.428 (3)
O7—C23	1.367 (2)	O5—C15	1.218 (3)
O7—C29	1.425 (3)	C1—C2	1.367 (3)
C22—C27	1.374 (3)	C1—C6	1.396 (3)
C22—C23	1.394 (3)	C12—C11	1.385 (3)
C16—C17	1.378 (3)	C12—H12A	0.9300
C16—C21	1.384 (3)	C10—C9	1.384 (3)
C19—C18	1.380 (3)	C10—C11	1.388 (3)
C19—C20	1.392 (3)	C10—C15	1.487 (3)
C19—C28	1.477 (3)	C11—H11A	0.9300
C20—C21	1.373 (3)	C3—C4	1.377 (3)
C20—H20A	0.9300	C3—C2	1.387 (3)
C23—C24	1.383 (3)	C3—C14	1.500 (3)
C21—H21A	0.9300	C6—C5	1.379 (3)
C27—C26	1.387 (3)	C2—H2A	0.9300
C27—H27A	0.9300	O4—C15	1.311 (3)
C24—C25	1.384 (3)	O4—H4	0.8200
C24—H24A	0.9300	C8—C9	1.379 (3)
C17—C18	1.382 (3)	C8—H8A	0.9300
C17—H17A	0.9300	C5—C4	1.384 (3)
O10—C28	1.207 (3)	C5—H5A	0.9300
O9—C28	1.311 (3)	C9—H9A	0.9300
O9—H9	0.8200	C4—H4A	0.9300
C18—H18A	0.9300	C14—O1	1.408 (3)
C26—C25	1.375 (3)	C14—H14A	0.9700
C26—C30	1.501 (3)	C14—H14B	0.9700
C25—H25A	0.9300	C13—H13A	0.9600
C29—H29A	0.9600	C13—H13B	0.9600
C29—H29B	0.9600	C13—H13C	0.9600
C29—H29C	0.9600	O6—C30	1.409 (3)
O3—C7	1.379 (3)	O6—H6	0.8200
O3—C1	1.393 (2)	O1—H1	0.8200
C7—C12	1.376 (3)	C30—H30A	0.9700
C7—C8	1.378 (3)	C30—H30B	0.9700
			
C16—O8—C22	117.53 (13)	O3—C1—C6	119.31 (19)
C23—O7—C29	117.67 (17)	C7—C12—C11	119.26 (18)
C27—C22—C23	121.00 (18)	C7—C12—H12A	120.4
C27—C22—O8	119.45 (18)	C11—C12—H12A	120.4
C23—C22—O8	119.49 (17)	C9—C10—C11	119.3 (2)
C17—C16—O8	123.43 (17)	C9—C10—C15	119.11 (19)
C17—C16—C21	120.64 (18)	C11—C10—C15	121.6 (2)
O8—C16—C21	115.91 (16)	C12—C11—C10	120.4 (2)
C18—C19—C20	118.90 (18)	C12—C11—H11A	119.8
C18—C19—C28	122.14 (18)	C10—C11—H11A	119.8
C20—C19—C28	118.92 (18)	C4—C3—C2	117.9 (2)
C21—C20—C19	120.65 (18)	C4—C3—C14	121.1 (2)
C21—C20—H20A	119.7	C2—C3—C14	120.9 (2)
C19—C20—H20A	119.7	O2—C6—C5	125.7 (2)
O7—C23—C24	125.23 (19)	O2—C6—C1	115.72 (19)
O7—C23—C22	116.22 (16)	C5—C6—C1	118.6 (2)
C24—C23—C22	118.54 (19)	C1—C2—C3	120.8 (2)
C20—C21—C16	119.57 (18)	C1—C2—H2A	119.6
C20—C21—H21A	120.2	C3—C2—H2A	119.6
C16—C21—H21A	120.2	C15—O4—H4	109.5
C22—C27—C26	120.5 (2)	C7—C8—C9	119.3 (2)
C22—C27—H27A	119.7	C7—C8—H8A	120.3
C26—C27—H27A	119.7	C9—C8—H8A	120.3
C23—C24—C25	119.8 (2)	O5—C15—O4	123.3 (2)
C23—C24—H24A	120.1	O5—C15—C10	122.5 (2)
C25—C24—H24A	120.1	O4—C15—C10	114.19 (19)
C16—C17—C18	119.21 (18)	C6—C5—C4	119.6 (2)
C16—C17—H17A	120.4	C6—C5—H5A	120.2
C18—C17—H17A	120.4	C4—C5—H5A	120.2
C28—O9—H9	109.5	C8—C9—C10	120.6 (2)
C19—C18—C17	120.98 (18)	C8—C9—H9A	119.7
C19—C18—H18A	119.5	C10—C9—H9A	119.7
C17—C18—H18A	119.5	C3—C4—C5	122.0 (2)
C25—C26—C27	118.34 (19)	C3—C4—H4A	119.0
C25—C26—C30	120.8 (2)	C5—C4—H4A	119.0
C27—C26—C30	120.7 (2)	O1—C14—C3	108.56 (18)
C26—C25—C24	121.80 (19)	O1—C14—H14A	110.0
C26—C25—H25A	119.1	C3—C14—H14A	110.0
C24—C25—H25A	119.1	O1—C14—H14B	110.0
O10—C28—O9	122.3 (2)	C3—C14—H14B	110.0
O10—C28—C19	123.6 (2)	H14A—C14—H14B	108.4
O9—C28—C19	113.99 (19)	O2—C13—H13A	109.5
O7—C29—H29A	109.5	O2—C13—H13B	109.5
O7—C29—H29B	109.5	H13A—C13—H13B	109.5
H29A—C29—H29B	109.5	O2—C13—H13C	109.5
O7—C29—H29C	109.5	H13A—C13—H13C	109.5
H29A—C29—H29C	109.5	H13B—C13—H13C	109.5
H29B—C29—H29C	109.5	C30—O6—H6	109.5
C7—O3—C1	118.79 (15)	C14—O1—H1	109.5
C12—C7—C8	121.1 (2)	O6—C30—C26	109.97 (19)
C12—C7—O3	123.66 (18)	O6—C30—H30A	109.7
C8—C7—O3	115.18 (19)	C26—C30—H30A	109.7
C6—O2—C13	117.22 (18)	O6—C30—H30B	109.7
C2—C1—O3	119.55 (19)	C26—C30—H30B	109.7
C2—C1—C6	121.01 (19)	H30A—C30—H30B	108.2
			
C16—O8—C22—C27	109.7 (2)	C7—O3—C1—C2	−109.8 (2)
C16—O8—C22—C23	−73.2 (2)	C7—O3—C1—C6	74.4 (2)
C22—O8—C16—C17	−24.5 (3)	C8—C7—C12—C11	−0.2 (3)
C22—O8—C16—C21	156.97 (17)	O3—C7—C12—C11	178.08 (18)
C18—C19—C20—C21	−1.2 (3)	C7—C12—C11—C10	0.8 (3)
C28—C19—C20—C21	176.59 (19)	C9—C10—C11—C12	−0.7 (3)
C29—O7—C23—C24	3.4 (3)	C15—C10—C11—C12	178.07 (18)
C29—O7—C23—C22	−177.65 (19)	C13—O2—C6—C5	5.0 (3)
C27—C22—C23—O7	−178.58 (17)	C13—O2—C6—C1	−174.72 (19)
O8—C22—C23—O7	4.4 (3)	C2—C1—C6—O2	−179.12 (18)
C27—C22—C23—C24	0.5 (3)	O3—C1—C6—O2	−3.4 (3)
O8—C22—C23—C24	−176.58 (17)	C2—C1—C6—C5	1.1 (3)
C19—C20—C21—C16	−0.7 (3)	O3—C1—C6—C5	176.89 (18)
C17—C16—C21—C20	2.4 (3)	O3—C1—C2—C3	−176.70 (18)
O8—C16—C21—C20	−179.01 (16)	C6—C1—C2—C3	−1.0 (3)
C23—C22—C27—C26	−0.7 (3)	C4—C3—C2—C1	0.2 (3)
O8—C22—C27—C26	176.39 (17)	C14—C3—C2—C1	−177.01 (18)
O7—C23—C24—C25	178.76 (19)	C12—C7—C8—C9	−0.3 (3)
C22—C23—C24—C25	−0.2 (3)	O3—C7—C8—C9	−178.76 (19)
O8—C16—C17—C18	179.35 (18)	C9—C10—C15—O5	8.9 (3)
C21—C16—C17—C18	−2.2 (3)	C11—C10—C15—O5	−169.9 (2)
C20—C19—C18—C17	1.4 (3)	C9—C10—C15—O4	−170.1 (2)
C28—C19—C18—C17	−176.28 (19)	C11—C10—C15—O4	11.1 (3)
C16—C17—C18—C19	0.2 (3)	O2—C6—C5—C4	179.73 (18)
C22—C27—C26—C25	0.6 (3)	C1—C6—C5—C4	−0.6 (3)
C22—C27—C26—C30	176.53 (19)	C7—C8—C9—C10	0.3 (3)
C27—C26—C25—C24	−0.3 (3)	C11—C10—C9—C8	0.2 (3)
C30—C26—C25—C24	−176.3 (2)	C15—C10—C9—C8	−178.65 (19)
C23—C24—C25—C26	0.1 (3)	C2—C3—C4—C5	0.4 (3)
C18—C19—C28—O10	170.7 (2)	C14—C3—C4—C5	177.58 (19)
C20—C19—C28—O10	−7.0 (4)	C6—C5—C4—C3	−0.2 (3)
C18—C19—C28—O9	−6.9 (3)	C4—C3—C14—O1	−91.9 (3)
C20—C19—C28—O9	175.4 (2)	C2—C3—C14—O1	85.2 (3)
C1—O3—C7—C12	11.8 (3)	C25—C26—C30—O6	87.2 (3)
C1—O3—C7—C8	−169.74 (19)	C27—C26—C30—O6	−88.7 (3)

### Hydrogen-bond geometry (Å, °)

**Table:**

*D*—H···*A*	*D*—H	H···*A*	*D*···*A*	*D*—H···*A*
O9—H9···O1	0.82	1.80	2.620 (2)	175
O4—H4···O6	0.82	1.84	2.652 (2)	1670
O6—H6···O10^i^	0.82	2.01	2.791 (3)	159
O1—H1···O5^ii^	0.82	1.89	2.706 (2)	172

Symmetry codes: (i) *x*, *y*, *z*+1; (ii) *x*, *y*, *z*−1.
